# Semiparametric confidence sets for cross-sectional and longitudinal neuroimaging

**DOI:** 10.1162/IMAG.a.965

**Published:** 2025-10-31

**Authors:** Xinyu Zhang, Kenneth Liao, Jakob Seidlitz, Maureen McHugo, Suzanne N. Avery, Anna Huang, Aaron Alexander-Bloch, Neil Woodward, Stephan Heckers, Simon Vandekar

**Affiliations:** Department of Biostatistics, Vanderbilt University, Nashville, TN, United States; Department of Psychiatry, University of Pennsylvania, Philadelphia, PA, United States; Department of Psychiatry, University of Colorado Anschutz Medical Campus, Aurora, CO, United States; Department of Psychiatry and Behavioral Sciences, Vanderbilt University Medical Center, Nashville, TN, United States; Department of Biostatistics, Vanderbilt University Medical Center, Nashville, TN, United States

**Keywords:** bootstrap, generalized estimating equation, multi-parameter analysis, neuroimaging data

## Abstract

The majority of neuroimaging inference focuses on hypothesis testing rather than effect estimation. With concerns about replicability, there is growing interest in reporting standardized effect sizes from neuroimaging group-level analyses. Confidence sets for effect sizes were recently developed for neuroimaging, but are restricted to simple univariate contrasts (e.g., one-sample or two-sample test Cohen’s d) and cross-sectional data. Thus, existing methods exclude increasingly common longitudinal associations of biological brain measurements with, potentially nonlinear, inter- and intra-individual variations in diagnosis, development, or symptoms. We use modern methods for confidence sets combined with a recently proposed robust effect size index to provide a very general approach and software for effect size confidence set inference in neuroimaging. Our method involves robust estimation of the effect size image and spatial and temporal covariance function based on generalized estimating equations. We use a nonparametric bootstrap to estimate the joint distribution of the robust effect size image across voxels to construct confidence sets. These confidence sets identify regions of the image where the lower or upper simultaneous confidence interval is above or below a given threshold with high probability. We evaluate the coverage and simultaneous confidence interval width of the proposed procedures using realistic simulations and perform longitudinal analyses of aging and diagnostic differences of cortical thickness in Alzheimer’s disease and diagnostic differences of resting-state hippocampal activity in psychosis. This comprehensive approach, along with the visualization functions integrated into the pbj R package, offers a robust tool for analyzing repeated neuroimaging measurements.

## 1 Introduction

Hypothesis testing using cluster extent inference is the most commonly used approach for investigating brain-behavior associations (Woo et al., 2014). This approach identifies regions of the brain image that are significantly associated with the non-brain phenotype by thresholding the statistical image for the voxel-level association above a given value, and, for each spatially contiguous “cluster”, computing a p-value representing the probability of observing a cluster of that size or larger if the phenotype was not associated with the brain measurement (Worsley et al., 1999). In response to long-held concerns about replicability, there has been growing interest in reporting standardized effect size estimates to characterize the strength of findings in neuroimaging (Bowring et al., 2019, 2021; Kang et al., 2024; Marek et al., 2022; Owens et al., 2021; S. N. Vandekar & Stephens, 2021). Standardized effect sizes are unitless indices quantifying the strength of an association (Kelley & Preacher, 2012). Because cluster extent inference is strictly focused on null hypothesis testing, it is not amenable to performing inference for effect sizes, as they are only non-zero when the null is false. In contrast to cluster extent inference, performing effect size-based statistical inference has several advantages (Bowring et al., 2021; Sommerfeld et al., 2018; S. N. Vandekar & Stephens, 2021). First, it provides a more nuanced understanding of the magnitude and practical significance of findings, beyond the arbitrary classification using hypothesis tests. Second, it facilitates meta-analyses and comparisons across studies, which is particularly important in evaluating and improving replicability (Zimmermann et al., 2005).

Confidence sets have long been an active area of research in statistics. A wide range of methodological approaches have been proposed, including Hausdorff distance-based methods (Chen et al., 2017; Seri & Choirat, 2004), bootstrap-based nonparametric developments (Mammen & Polonik, 2013), and subsequent geometric refinements (Qiao & Polonik, 2019). Related work has also appeared in econometrics (Bugni, 2010), geostatistics (French, 2014; French & Hoeting, 2016; French & Sain, 2013), and climate applications (Sommerfeld et al., 2018). More recently, Ren et al. (2024) proposed an elegant approach to construct confidence sets from simultaneous confidence intervals. In neuroimaging, where these ideas have been adapted to standardized or unstandardized effect sizes, the existing methods have been developed only for simple one-sample raw effect sizes (Bowring et al., 2019) and two-sample Cohen’s d (Bowring et al., 2021), and have not been generalized to multi-group comparisons or nonlinear associations with age or symptom severity, which are often used for modeling the complex associations in brain-behavior associations (Grabell et al., 2018; Zhou et al., 2023). In addition, they do not explicitly account for the temporal covariance structure in the data, thus making their application to the longitudinal setting difficult. Longitudinal analyses are increasingly valued for its ability to study within-subject associations (Kang et al., 2024; Van Os et al., 1999).

Here, we combine recently developed statistical methods (Guillaume et al., 2014; Jones et al., 2025; Kang et al., 2023; Liang & Zeger, 1986; Ren et al., 2024) into a unique framework that addresses the limitations of existing confidence set approaches for neuroimage analysis. First, our approach allows for the construction of confidence sets for a robust effect size index (RESI) that is very general, so includes inference for multi-dimensional parameters needed to study multi-group and nonlinear associations in brain-related disorders (S. Vandekar et al., 2020). Second, the existing methods (Bowring et al., 2021) are limited to cross-sectional settings; our framework integrates generalized estimating equations (GEEs) to accommodate within-subject dependence in longitudinal neuroimaging data (Guillaume et al., 2014; Liang & Zeger, 1986). Third, we use a recently developed approach for confidence set estimation based on simultaneous confidence intervals (Ren et al., 2024; Telschow et al., 2024). We demonstrate the utility of our method through simulations, showing its robust performance across a range of sample sizes (25-500), and apply it to study effect sizes in longitudinal analyses of regional cortical thickness differences in Alzheimer’s disease and resting-state activity in psychosis-spectrum disorders. Our results highlight the potential of this approach to provide new insights beyond cluster extent inference. The methods presented in this paper are available in the pbj R package (https://github.com/statimagcoll/pbj), and the code for the analyses can be found here (https://github.com/statimagcoll/Confidence_Sets.git).

## 2 Motivating Data

### 2.1 The ADNI dataset analysis

The Alzheimer’s Disease Neuroimaging Initiative (ADNI) is a longitudinal, multi-center, observational study. Patients were recruited across North America from 2004 through the present. Diagnostic categories include control (CN), some memory concerns (SMC), early and late mild cognitive impairment (EMCI, LMCI), and Alzheimer’s Disease (AD). Average cortical thickness within regions of the Desikan-Killiany atlas was estimated using the Freesurfer cortical parcellation pipeline. Quality control was performed using a combination of expert visual curation and automated metrics of image quality (Bethlehem et al., 2022). ADNI T1-weighted images were processed in-house using the FreeSurfer v6 (FS6_T1) pipeline and acquired with the ADNI MPRAGE protocol (Bethlehem et al., 2022, ST 1.1). Detailed acquisition parameters are provided in Jack Jr. et al. (2008). Participant demographic information can be found in Table 1. Our goal in the ADNI analysis is to characterize effect sizes for the associations of age and diagnosis with regional measurements of cortical thickness in the Desikan-Killiany atlas (Desikan et al., 2006).

**Table 1.** Participant demographic information by diagnosis in the ADNI dataset.

	CN	SMC	EMCI	LMCI	AD
	(n = 414)	(n = 102)	(n = 308)	(n = 558)	(n = 327)
Sex					
Female	206	59	138	216	148
	(49.8%)	(57.8%)	(44.8%)	(38.7%)	(45.3%)
Male	208	43	170	342	179
	(50.2%)	(42.2%)	(55.2%)	(61.3%)	(54.7%)
Age (years)					
Mean	74.8	72.0	71.1	74.0	74.9
(SD)	(5.77)	(5.55)	(7.46)	(7.49)	(7.87)
Median	74.1	71.2	70.8	74.4	75.4
[Min, Max]	[56.2, 89.7]	[59.7, 90.2]	[55.0, 88.7]	[54.4, 91.5]	[55.1, 91.0]
Session					
1	413 (26.0%)	102 (76.1%)	306 (27.0%)	556 (27.9%)	327 (43.3%)
2	372 (23.4%)	20 (14.9%)	272 (24.0%)	471 (23.6%)	232 (30.7%)
3	337 (21.2%)	6 (4.5%)	249 (22.0%)	394 (19.8%)	175 (23.2%)
4	241 (15.2%)	6 (4.5%)	202 (17.8%)	275 (13.8%)	21 (2.8%)
5	146 (9.2%)	0 (0%)	97 (8.6%)	156 (7.8%)	0 (0%)
6	57 (3.6%)	0 (0%)	8 (0.7%)	71 (3.6%)	0 (0%)
7	15 (0.9%)	0 (0%)	0 (0%)	47 (2.4%)	0 (0%)
8	6 (0.4%)	0 (0%)	0 (0%)	11 (0.6%)	0 (0%)
9	2 (0.1%)	0 (0%)	0 (0%)	7 (0.4%)	0 (0%)
10	0 (0%)	0 (0%)	0 (0%)	4 (0.2%)	0 (0%)

n denotes the total number of participants.

CN = Cognitively Normal, SMC = Significant Memory Concern, EMCI = Early Mild Cognitive Impairment, LMCI = Late Mild Cognitive Impairment, AD = Alzheimer’s Disease.

### 2.2 Early psychosis analysis in the PGPP

The Psychiatric Genotype-Phenotype Project (PGPP) includes data collected at the Vanderbilt Psychiatric Hospital between May 2013 and February 2018 for a prospective 2-year longitudinal study, and was approved by the IRB at Vanderbilt University Medical Center. The inclusion criteria for patients were a diagnosis of a non-affective psychotic disorder with a duration of psychosis of less than 2 years. Exclusion criteria for all participants included significant head injury, major medical illnesses, pregnancy, presence of metal, claustrophobia, and current substance abuse or dependence within the past month prior to study enrollment. Participant demographic and clinical characteristics are listed in Table 2 (McHugo et al., 2022).

**Table 2.** Demographic and clinical characteristics by diagnosis for participants in the PGPP study.

	Early psychosis	Healthy control
	(n = 59)	(n = 67)
Age		
Mean (SD)	21.4 (3.93)	21.8 (2.84)
Median [Min, Max]	20.0 [16.0, 39.0]	21.0 [17.0, 29.0]
Sex		
Female	13 (22.0%)	17 (25.4%)
Male	46 (78.0%)	50 (74.6%)
Race		
Asian	1 (1.7%)	1 (1.5%)
Black or African American	13 (22.0%)	10 (14.9%)
White	45 (76.3%)	53 (79.1%)
Other	0 (0%)	3 (4.5%)
Time		
Baseline	59 (53.6%)	67 (56.8%)
Follow-up	51 (46.4%)	51 (43.2%)

n denotes the total number of participants.

Our goal in these analyses was to investigate the hypothesis of progressive hippocampal excitation/inhibition (E/I) imbalance in the early stages of psychosis (McHugo et al., 2022). E/I imbalance was operationalized as fractional amplitude of low-frequency fluctuations (fALFF) in the hippocampus. fALFF is a voxel-level frequency-domain scalar summary of brain activity during a resting-state functional MRI scan.

After registration of the fMRI time series data to the MNI 2 mm template, fALFF was calculated using AFNI’s 3dRSFC tool (Taylor & Saad, 2013) as the average of the power in the range 0.01–0.1 Hz relative to the entire frequency spectrum, and normalized by dividing by the mean fALFF within the whole brain at each voxel (McHugo et al., 2022). Fractional ALFF is less susceptible than standard ALFF to motion, physiological artifacts, and site differences compared to standard ALFF, making it a more reliable indicator of neural activity in clinical populations (Yan et al., 2013; Zou et al., 2008). In this analysis, the spatial index v corresponds to individual voxels within the hippocampus, consisting of 1,055 voxels in total. Data from nine participants were excluded for imaging data quality control or due to ineligible diagnoses at follow-up, yielding 59 patients and 67 healthy control participants. Details of image processing, data quality control, and exclusionary criteria are available in prior work (McHugo et al., 2022).

## 3 Methods

### 3.1 Notation and background

Our confidence set method is based on the general linear model. We let $Y_{i}\left(v\right)\in {\mathbb{R}}^{n_{i}}$ denote the outcome vector image of $n_{i}$ repeated observations of brain measurements for i=1, 2,⋯,n independent subjects. These images are indexed by the spatial location, v∈V , where V denotes the bounded space of the brain or the cortical sheet, and we assume the data are registered to a template space. For notational simplicity, we refer to v as a “voxel” throughout the paper, although in practice v can also represent a parcellation unit or an atlas region. For each location v, we assume the model

$$\begin{array}{cc} Y_{i}\left(v\right) & =X_{0,i}\alpha \left(v\right)+X_{1,i}\beta \left(v\right)+E_{i}\left(v\right)\\ & =X_{i}\zeta \left(v\right)+E_{i}\left(v\right) \end{array}$$ (1)

where $X_{0,i}\in {\mathbb{R}}^{n_{i}\times m_{0}}$ is a matrix of nuisance covariates including the intercept, $X_{1,i}\in {\mathbb{R}}^{n_{i}\times m_{1}}$ is a matrix of variables of interest (such as multi-group diagnosis or age fit with splines), $m=m_{0}+m_{1}$, and $X_{i}=\left[X_{0,i},X_{1,i}\right]\in {\mathbb{R}}^{n_{i}\times m}$ ; α(v) and β(v) are parameter image vectors that take values in ${\mathbb{R}}^{m_{0}}$ and ${\mathbb{R}}^{m_{1}}$, respectively, and $\zeta \left(v\right)={\left[\alpha {\left(v\right)}^{T},\beta {\left(v\right)}^{T}\right]}^{T}\in {\mathbb{R}}^{m}$; $E_{i}\left(v\right)\in {\mathbb{R}}^{n_{i}}$ is an error term vector with mean zero and spatial covariance matrix $\Sigma_{i}\left(v,w\right)=\text{Cov}\left\{E_{i}\left(v\right),E_{i}\left(w\right)\right\}<\infty$ for any two voxels v and w. This covariance describes the dependence between repeated imaging measurements over time and spatially within the image. The multilevel and spatial aspects of the data make them susceptible to unknown multivariate heteroskedasticity: $\Sigma_{i}\left(v,w\right)\neq \Sigma_{j}\left(v,w\right)$ for i≠j . To allow matrix notation, let $N=\sum_{i}\;n_{i}$ be the number of all observations across n independent participants. We define the design matrices as follows: $X_{0}={\left[X_{0,1}^{T},\cdots ,X_{0,n}^{T}\right]}^{T}\in {\mathbb{R}}^{N\times m_{0}}$, $X_{1}={\left[X_{1,1}^{T},\cdots ,X_{1,n}^{T}\right]}^{T}\in {\mathbb{R}}^{N\times m_{1}}$, $X=\left[X_{0},X_{1}\right]\in {\mathbb{R}}^{N\times m}$ . Similarly, we define the full response vector at location v as $Y\left(v\right)={\left[Y_{1}^{T}\left(v\right),\cdots ,Y_{n}^{T}\left(v\right)\right]}^{T}\in {\mathbb{R}}^{N}$, and the corresponding error vector as $E\left(v\right)={\left[E_{1}^{T}\left(v\right),\cdots ,E_{n}^{T}\left(v\right)\right]}^{T}\in {\mathbb{R}}^{N}$ with covariance $\Sigma \left(v,w\right)=\text{diag }\left\{\Sigma_{1}\left(v,w\right),\ldots ,\Sigma_{n}\left(v,w\right)\right\}\in {\mathbb{R}}^{N\times N}$ .

For estimation using GEE, let $V_{w}\left(\gamma \right)\in {\mathbb{R}}^{N\times N}$ denote the block diagonal temporal working covariance matrix for all subjects, which we assume does not depend on the location in the image. γ is the working correlation parameter that controls the temporal covariance for measurements from the same participant. Let $R_{0}$ and R denote the symmetric N×N residual forming matrices for $X_{0}$ and X, respectively (see Supplementary Section S1.2 for their derivation). To ease notations below, we do not explicitly include the dependence of $V_{w}$ on γ.

In this paper, we refer to the sample size as the number of independent subjects n, despite each subject having multiple longitudinal observations. All vectors are, by default, treated as column vectors.

### 3.2 Parameter estimation and test statistic

It is critical to account for repeated observations from the same participant when estimating β(v) and constructing confidence intervals. Prior estimating equation methods for neuroimaging data only used an independence working covariance structure due to the high computational demand of fitting over 100,000 voxel-level models (Guillaume et al., 2014). We perform estimation using working independence and exchangeable covariance structures using a one-step least squares estimator (Lipsitz et al., 2017). The one-step estimation speeds computation for the covariance parameters, which typically require iterative estimation in GEEs. The test statistic image is derived using robust covariance estimation in the context of GEEs, treating each voxel separately (Huber, 1964; Liang & Zeger, 1986; White, 1980). Dependence among voxels is incorporated in preprocessing (via spatial smoothing) and in the effect size analysis using a joint bootstrap.

The estimating equation is derived based on model (1) as the score equation under an exponential family model with identity link (Liang & Zeger, 1986), which does not require Gaussian assumptions (see Supplementary Section S1 for the derivation). This yields the GEE

$$\Psi \left(\zeta ,v;Y\right)=X^{T}V_{w}^{-1}\left\{Y\left(v\right)-X\zeta \left(v\right)\right\}=0.$$ (2)

When the variance is known, solving this estimating equation gives the unbiased least squares estimator for β(v) that corresponds to the variables of interest,

$$\hat{\beta }\left(v\right)={\left\{X_{1}^{T}V_{w}^{-1/2}R_{0}V_{w}^{-1/2}X_{1}\right\}}^{-1}\;X_{1}^{T}V_{w}^{-1/2}R_{0}V_{w}^{-1/2}Y\left(v\right).$$ (3)

More details on this derivation are given in Supplementary Section S1. To obtain the one-step estimator for the exchangeable covariance structure, $V_{w}$ is taken to be identity to obtain an initial estimate of β(v); then, the correlation parameter, $\hat{\gamma }$, is estimated conditional on the initial estimate of β(v); and the final estimator, $\hat{\beta }\left(v\right)$, is obtained by plugging in $\hat{\gamma }$ into (3) (Supplementary Section S2).

The robust estimator for the asymptotic covariance of $\hat{\beta }\left(v\right)$, denoted by $\Sigma_{\beta }\left(v,w\right)$, is derived by a first-order Taylor expansion (Liang & Zeger, 1986). We estimate the asymptotic covariance with ${\hat{\Sigma }}_{\beta }\left(v,w\right)={\hat{A}}_{\beta }^{-1}{\hat{\Omega }}_{\beta }\left(v,w\right){\hat{A}}_{\beta }^{-1}$ , where

$$\{\begin{array}{c} {\hat{A}}_{\beta }=X_{1}^{T}V_{w}^{-1/2}\left(\hat{\gamma }\right)R_{0}V_{w}^{-1/2}\left(\hat{\gamma }\right)X_{1}\\ {\hat{\Omega }}_{\beta }\left(v,w\right)=X_{1}^{T}V_{w}^{-1/2}\left(\hat{\gamma }\right)R_{0}\hat{\Sigma }\left(v,w\right)R_{0}V_{w}^{-1/2}\left(\hat{\gamma }\right)X_{1}, \end{array}$$ (4)

and $\hat{\Sigma }\left(v,w\right)$ is block-diagonal with elements ${\hat{\Sigma }}_{i}\left(v,w\right)$, as defined in the Supplementary Equation (S2) (Bell & McCaffrey, 2002; MacKinnon et al., 2023).

Given Equations (3) and (4), the Wald test statistic can be constructed for a reference null hypothesis $H_{0}\left(v\right)\;:\beta \left(v\right)=\beta_{0}\left(v\right)$. When the variance is known, under mild regularity conditions such as those stated in Theorems 5.21 and 5.23 of Van der Vaart (2000), this test statistic asymptotically follows a non-central chi-squared distribution with $m_{1}$ degrees of freedom, where the non-centrality parameter is

$$n{\left\{\beta \left(v\right)-\beta_{0}\left(v\right)\right\}}^{T}\Sigma_{\sqrt{n}\beta }^{-1}\left(v\right)\left\{\beta \left(v\right)-\beta_{0}\left(v\right)\right\},$$

with $\Sigma_{\sqrt{n}\beta }\left(v\right)=\text{Cov}\left\{\sqrt{n}\hat{\beta }\left(v\right)\right\}$. We shall not detail these conditions here, but note that further detail and discussion can be found in S. Vandekar et al. (2020), Appendix. When the variance of the parameter is unknown, the test statistic image is

$$\begin{array}{cc} & T_{m_{1}}^{2}\left(v\right)=n{\left\{\hat{\beta }\left(v\right)-\beta_{0}\left(v\right)\right\}}^{T\;}{\hat{\Sigma }}_{\sqrt{n}\beta }^{-1}\left(v\right)\left\{\hat{\beta }\left(v\right)-\beta_{0}\left(v\right)\right\}, \end{array}$$ (5)

which can be used to compute the RESI (Jones et al., 2025).

### 3.3 RESI definition, estimator, and bootstrap

The RESI is based on M-estimators, such as those obtained from a GEE. Our reason for using the RESI as a standardized effect size index is that it is robust, generalizable, and easily interpretable across a wide range of models and study designs (S. Vandekar et al., 2020).

Detailed definitions and theory are given in prior work (Jones et al., 2025; Kang et al., 2023; S. Vandekar et al., 2020). To summarize, for a given voxel, v, the RESI $S_{\beta }\left(v\right)$ is defined as the square root of the non-centrality parameter of the chi-squared statistic mentioned above, after removing the scaling by the sample size n. That is,

$$S_{\beta }\left(v\right)={\left[{\left\{\beta \left(v\right)-\beta_{0}\left(v\right)\right\}}^{T}\Sigma_{\sqrt{n}\beta }^{-1}\left(v\right)\left\{\beta \left(v\right)-\beta_{0}\left(v\right)\right\}\right]}^{1/2}.$$ (6)

A consistent estimator of the RESI is obtained from the test statistic as (López-Blázquez, 2000; S. Vandekar et al., 2020)

$${\hat{S}}_{\beta }\left(v\right)={\left\{\text{max}\left(0,\frac{T_{m_{1}}^{2}\left(v\right)-m_{1}}{n}\right)\right\}}^{1/2}.$$ (7)

We use a nonparametric bootstrap procedure to construct confidence sets for the RESI. The percentile-normal and percentile-t bootstraps are commonly used methods for confidence intervals (Hall, 1992, p. 86-87, 92–93). In general, the percentile-t nonparametric bootstrap has better small sample performance than the percentile-normal bootstrap because it accounts for the estimation of the variance of the parameter estimator and has smaller higher-order error terms than the percentile-normal bootstrap (Hall, 1992, p. 86-87, 92–93). However, the percentile-t bootstrap requires an estimate of the variance of the RESI estimator in each bootstrap (Hall, 1992, p. 86-87). Because deriving the variance of (7) is challenging due to the max operator, we instead derive the asymptotic variance of

$${\tilde{S}}_{\beta }\left(v\right)={\left(\frac{T_{m_{1}}^{2}\left(v\right)}{n}\right)}^{1/2},$$

assuming the data are normally distributed and using the delta method (Supplementary Section S3),

$$\sigma^{2}\left(v\right)=\;\text{Var }\left\{\sqrt{n}{\tilde{S}}_{\beta }\left(v\right)\right\}=S_{\beta }^{2}\left(v\right)/2+1.$$ (8)

We use a plug-in estimator for the square root of this variance term to normalize the effect size estimator in each bootstrap. Although the normality assumption is likely violated, performing this normalization may improve the performance of the bootstrap if it is approximately accurate (Hall, 1992, p. 196). This improvement stems from its studentized statistic $\left(\hat{\theta }-\theta )/\hat{se}(\hat{\theta }\right)$ , which more closely approximates a pivotal quantity than the original statistic $\hat{\theta }$. This makes its sampling distribution less dependent on population shape (e.g., skewness, kurtosis), leading to better higher-order properties.

This block-resampling approach assumes independence and exchangeability across subjects and preserves the within-subject spatiotemporal correlation structure. Note the length of $\left(Y^{b}\left(v\right),X^{b}\right)$ could vary across bootstrap replicates. We evaluate the percentile-t bootstrap and the percentile-normal bootstrap, where standard deviation, ${\hat{\sigma }}^{b}\left(v\right)$, is taken to be 1, in simulations below.

### 3.4 Confidence sets

Confidence sets define a collection of voxels or regions that form inner (or outer) spatial regions used to make probabilistic statements about brain regions where the true effect size is larger (or smaller) than a given non-zero effect size threshold (Aitchison, 1964; Bowring et al., 2021; Sommerfeld et al., 2018). For a given effect size threshold s and confidence level 1−∈ , the inner and outer confidence sets are defined as ${\text{CS}}_{\text{in}}\left(s,\in \right)\subset V$ and ${\text{CS}}_{\text{out}}\left(s,\in \right)\subset V$ that satisfy (Telschow et al., 2024)

$$\mathbb{P}({\text{CS}}_{\text{in}}\left(s,\in \right)\subset \{v\;:S_{\beta }\left(v\right)>s\}\subset {\text{CS}}_{\text{out}}\left(s,\in \right))=1-\in .$$ (9)

Prior work showed that 1−∈ level confidence sets can be derived from simultaneous confidence intervals (SCIs) that are valid for all effect size thresholds, s in (9) (Ren et al., 2024; Telschow et al., 2024). Let ${\hat{B}}_{l}\left(v,\in \right)$ and ${\hat{B}}_{u}\left(v,\in \right)$ denote the estimated lower and upper SCIs functions at level ϵ such that:

$$\mathbb{P}\left[{\hat{B}}_{l}\left(v,\epsilon \right)\leq S_{\beta }\left(v\right)\leq {\hat{B}}_{u}\left(v,\epsilon \right):\forall v\in V\right]=1-\epsilon ,$$

SCIs control the coverage of CIs simultaneously over all voxels or atlas regions. We use their result to construct confidence sets for the RESI in longitudinal GEE, combining Algorithm 1 above with Algorithm 2 to obtain SCIs.

**Table:** **Algorithm 1:** Percentile-t bootstrap

1. Initialize integer number of bootstraps B, number of subjects n, and let $\left(Y_{i}\left(v\right),X_{i}\right)$ be as defined above (Section 3.1). 2. **for** b←1 **to** B **do** (1): Obtain $\left(Y^{b}\left(v\right),X^{b}\right)$ by block sampling with replacement at the subject level. Specifically, sample n subjects with replacement. Each “block” is the full set of observations from a single subject and is treated as a new subject with a unique identifier. (2): Compute the test statistic ${\left(T_{m_{1}}^{2}\right)}^{b}\left(v\right)$ using formula (3), (4), and (5), and ${\hat{S}}_{\beta }^{b}\left(v\right)$ using formula (7). (3): Compute the normalized estimated RESI $N^{b}\left(v\right)=\frac{{\hat{S}}_{\beta }^{b}\left(v\right)-{\hat{S}}_{\beta }\left(v\right)}{{\hat{\sigma }}^{b}\left(v\right)}$, where ${\hat{\sigma }}^{b}\left(v\right)$ is obtained with a plug-in estimator using (8). **end** 3. Return the array of estimated RESI, ${\left\{N^{b}\left(v\right)\right\}}_{b\leq B}$ as the bootstrap estimate of the distribution for $\frac{{\hat{S}}_{\beta }\left(v\right)-S_{\beta }\left(v\right)}{\hat{\sigma }\left(v\right)}$.

**Table:** **Algorithm 2:** 1-∈ level RESI confidence set

1. Let effect size threshold s>0 and ∈∈(0, 1) be given. 2. Sample normalized test statistics ${\left\{N^{b}\left(v\right)\right\}}_{b\leq B}$ using Algorithm 1. 3. Set $U^{b}={\text{max}}_{v}\;N^{b}\left(v\right)$ and $L^{b}={\text{min}}_{v}\;N^{b}\left(v\right)$. 4. Set U(∈) as the $1-\frac{\in }{2}$ empirical quantile of ${\left\{U^{b}\right\}}_{b\leq B}$ and L(∈), as the $\frac{\in }{2-\in }$ empirical quantile of the subset ${\left\{L^{b}|U^{b}\leq U\left(\in \right)\right\}}_{b\leq B}$ . 5. Return the estimated 1−∈ level SCIs $$\left[{\hat{B}}_{l}\left(v,\in \right),{\hat{B}}_{u}\left(v,\in \right)]=[\hat{S}\left(v\right)-U\left(\in \right)*\hat{\sigma }\left(v\right),\;\hat{S}\left(v\right)-L\left(\in \right)*\hat{\sigma }\left(v\right)\right],$$ where $\hat{\sigma }\left(v\right)$ is plug-in estimate of (8). 6. Threshold the lower SCI by s to obtain the inner confidence set, and threshold the upper SCI by s to obtain the outer confidence set $$\begin{array}{ccc} {\text{CS}}_{\text{in}}\left(s,\in \right) & =\{v|{\hat{B}}_{l}\left(v,\in \right)>s\} & \\ {\text{CS}}_{\text{out}}\left(s,\in \right) & =\{v|{\hat{B}}_{u}\left(v,\in \right)>s\}. & \end{array}$$

We define two key regions based on the SCIs and confidence set:

- **Acceptance set**: ${\text{CS}}_{\text{in}}\left(s,\in \right)=\{v|{\hat{B}}_{l}\left(v,\in \right)>s\}$ , voxels where the effect size is likely above the threshold s
- **Rejection set**: ${\text{CS}}_{\text{out}}{\left(s,\in \right)}^{C}=\{v|{\hat{B}}_{u}\left(v,\in \right)<s\}$ , voxels where the effect size is likely below the threshold s

These sets offer an effect–size–based framework that parallels traditional hypothesis testing in interpretation. The threshold s plays a role analogous to a null value, and the acceptance set highlights where the effect size likely exceeds this threshold, similar to significant voxels using p-values. In contrast, the rejection set includes locations where the effect is likely negligible in reference to the threshold, aiding interpretation and follow-up analysis. While such regions can also be identified via strong FWER-controlling hypothesis testing procedures (Telschow et al., 2024), confidence sets offer a geometric and threshold-free perspective, particularly well-suited to spatial inference problems.

## 4 Simulation

### 4.1 Simulation methods

We use simulations to evaluate the performance of the confidence set procedures under different scenarios. Because the confidence sets we use are based on SCIs (Ren et al., 2024), we evaluate the bias, simultaneous and voxel-wise coverage, and width of the SCIs in 500 replications. SCIs are constructed at the 95% confidence level. In this simulation study, the spatial index v corresponds to individual voxels. The study mask used in the simulation was obtained by restricting the MNI gray matter mask to the 46th axial slice of PGPP data, which contains 1,845 voxels, to reduce computational burden. Slice 46 was selected as it is approximately the middle slice out of the 91 total axial slices, providing a representative cross-section of the brain.

For the simulation, we simulate a longitudinal regression model for participant i,

$$Y_{ij}\left(v\right)=\beta_{0}+\beta_{1}\left(v\right){\text{age}}_{ij}+\beta_{2}\left(v\right){\text{sex}}_{i}+E_{ij}\left(v\right).$$

The number of measurements per subject is randomly generated from a uniform distribution ranging between 1 and 3. For each subject i, we first sample a subject-specific mean age from a uniform distribution between 20 and 70. Then, for each observation $j=1,\;2,\cdots ,n_{i}$, the covariate ${\text{age}}_{ij}$ is generated from a normal distribution with that subject-specific mean and a fixed variance of 1. ${\text{sex}}_{i}$ (fixed within-subject) is sampled from a Bernoulli distribution with a probability of 0.5. The true value for $\beta \left(v\right)={\left[\beta_{0},\beta_{1}^{T}\left(v\right),\beta_{2}^{T}\left(v\right)\right]}^{T}$ is derived from voxel-wise coefficient estimates obtained using longitudinal fMRI data from the PGPP dataset. We extract signals from a masked region in the 46th axial slice and fit a marginal model to each voxel using generalized estimating equations (GEE), adjusting for age, sex, group, and time, including a group-by-time interaction. The estimated age and sex effects are then scaled by 0.7 and 2, respectively, to define $\beta_{1}\left(v\right)$ and $\beta_{2}\left(v\right)$. The intercept $\beta_{0}$ is set to be 1. The PGPP dataset provides realistic spatial patterns and effect heterogeneity across voxels, making it suitable for defining the simulation ground truth.

For the error term, we use a spatio-temporal covariance structure that is the Kronecker product of separate spatial and temporal covariances (Chi, 2016). Let $R_{s}\left(v\right)\in {\mathbb{R}}^{N_{\text{PGPP}}}$ represent the residuals vector at location v estimated from the study mask using GEE (and scaled by $\sqrt{n-\text{df}}$ ), where $N_{\text{PGPP}}=228$ is the total number of observations in the PGPP data, n is the number of independent participants in the simulation setting, and df is the model degrees of freedom. $v=1,\;2,\ldots ,N_{V}$ where $N_{V}=1,845$ is the number of voxels in the study mask. The true spatial covariance between locations v and w, denoted $C_{s}\left(v,w\right)$, is a scalar quantity, and this scalar covariance is the same across all participants. To simulate a temporal covariance among repeated observations within the participant i, we let $C_{t,i}\in {\mathbb{R}}^{n_{i}\times n_{i}}$ denote the temporal correlation structure that we allow to be independent, exchangeable, or AR1, where $n_{i}$ is the number of observations for the participant i in our simulation. This structure can be represented as $C_{t,i}=R_{t,i}R_{t,i}^{T}$, where $R_{t,i}\in {\mathbb{R}}^{n_{i}\times n_{i}}$ is derived through Cholesky decomposition. We construct the spatio-temporal error term at location $v=1,\;2,\ldots ,N_{V}$ for participant i, by

$$E_{i}\left(v\right)=R_{t,i}Z_{i}R_{s}\left(v\right)\in {\mathbb{R}}^{n_{i}},$$

where $Z_{i}\in {\mathbb{R}}^{n_{i}\times N_{\text{PGPP}}}$ is a matrix of independent standard normal random variables. The covariance for subject i at voxels v and w is

$$\text{Cov}\left\{E_{i}\left(v\right),E_{i}\left(w\right)\right\}=C_{s}\left(v,w\right)\times C_{t,i}\in {\mathbb{R}}^{n_{i}\times n_{i}},$$

where Cov is the covariance operator. The full covariance structure across all voxels and all subjects is block-diagonal, with each block corresponding to subject i and having size $n_{i}N_{V}\times n_{i}N_{V}$. The full covariance takes the Kronecker product form: $\Sigma =C_{s}⊗C_{t}\in {\mathbb{R}}^{\left(NN_{V}\right)\times \left(NN_{V}\right)}$. While we rely on spatio-temporal separability for simulating data, the theoretical results hold under general covariance structures. We evaluate the procedure under a non-separable covariance structure in Supplementary Section S5.

We vary the sample size n∈{25, 50, 100, 150, 200, 300, 400, 500} across the six combinations of three true temporal covariances (independent, exchangeable, and AR1) and two working covariances (independent and exchangeable), and estimate the confidence sets using 1,000 bootstrap samples and the two Bootstrap methods mentioned in Section 3.3.

The true effect size depends on the working covariance structure as it changes the asymptotic covariance of the estimator for β(v). The asymptotic covariance $\Sigma_{\sqrt{n}\beta }\left(v\right)$ in Equation (6) is

$$\text{Cov}\left\{\sqrt{n}\hat{\beta }\left(v\right)\right\}=\underset{n\to \infty }{\text{lim}}nE\left\{{\left(X^{T}C_{work}^{-1}X\right)}^{-1}\right\}\;E\left(X^{T}C_{work}^{-1}C_{true}C_{work}^{-1}X\right)\;E\left\{{\left(X^{T}C_{work}^{-1}X\right)}^{-1}\right\}\;\sigma^{2}\left(v\right),$$

where $C_{work}$ is the working correlation, $C_{true}$ is the true correlation, and $\sigma^{2}\left(v\right)$ is the empirical variance of the residuals from the model fit in the PGPP data (Supplementary Section S4). To determine this value computationally, we use Monte Carlo integration with 1,000 replicates with 3,000 subjects to approximate this asymptotic value. The subject-level covariance is estimated using a jackknife-based estimator (see Equation (S2)), which accounts for heteroskedasticity in the data.

### 4.2 Simulation results

**Fig. 1.**
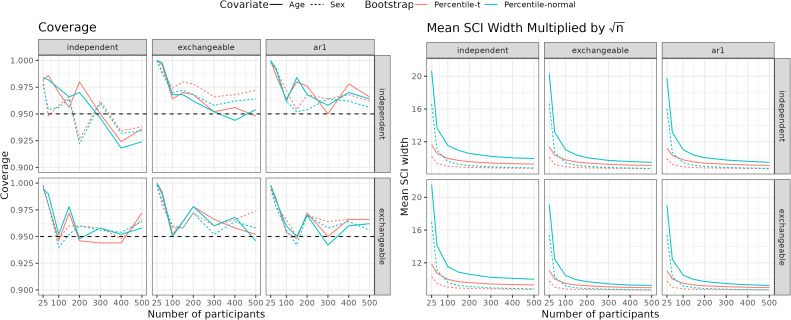
Simulation results for simultaneous coverage (left) and $\sqrt{n}$ times mean width of the simultaneous confidence intervals (right) used to construct confidence sets. The panel columns indicate the true covariance structures (independent, exchangeable, and AR1), and the panel rows are working covariance structures (independent and exchangeable) for different sample sizes (25-500). Each scenario considers two covariates (age and sex) and two Bootstrap methods (percentile-t and percentile-normal).

We ran simulations to assess the simultaneous coverage of the SCIs across all voxels and the mean width of the confidence intervals (averaged across all voxels) used to construct the confidence sets. The simultaneous coverage is above the nominal level and decreases to the nominal level as the sample size increases, regardless of the true or working covariance structure (Fig. 1, left). To facilitate meaningful comparison of SCIs width across sample sizes, we multiply the width by $\sqrt{n}$ so that any changes represent small sample bias. Confidence interval width decreases with increasing sample size, indicating positive bias of the standard error in small samples. The percentile-t bootstrap has substantially smaller confidence intervals in small samples across all scenarios (Fig. 1, right). Because the percentile-t bootstrap produces narrower SCIs and has equivalent coverage, it is superior to the percentile-normal bootstrap. See Supplementary Section S6 for plots of the median and max CI width. Results with a non-separable covariance structure can be found in the Supplementary Section S5 and are consistent with the findings here.

**Fig. 2.**
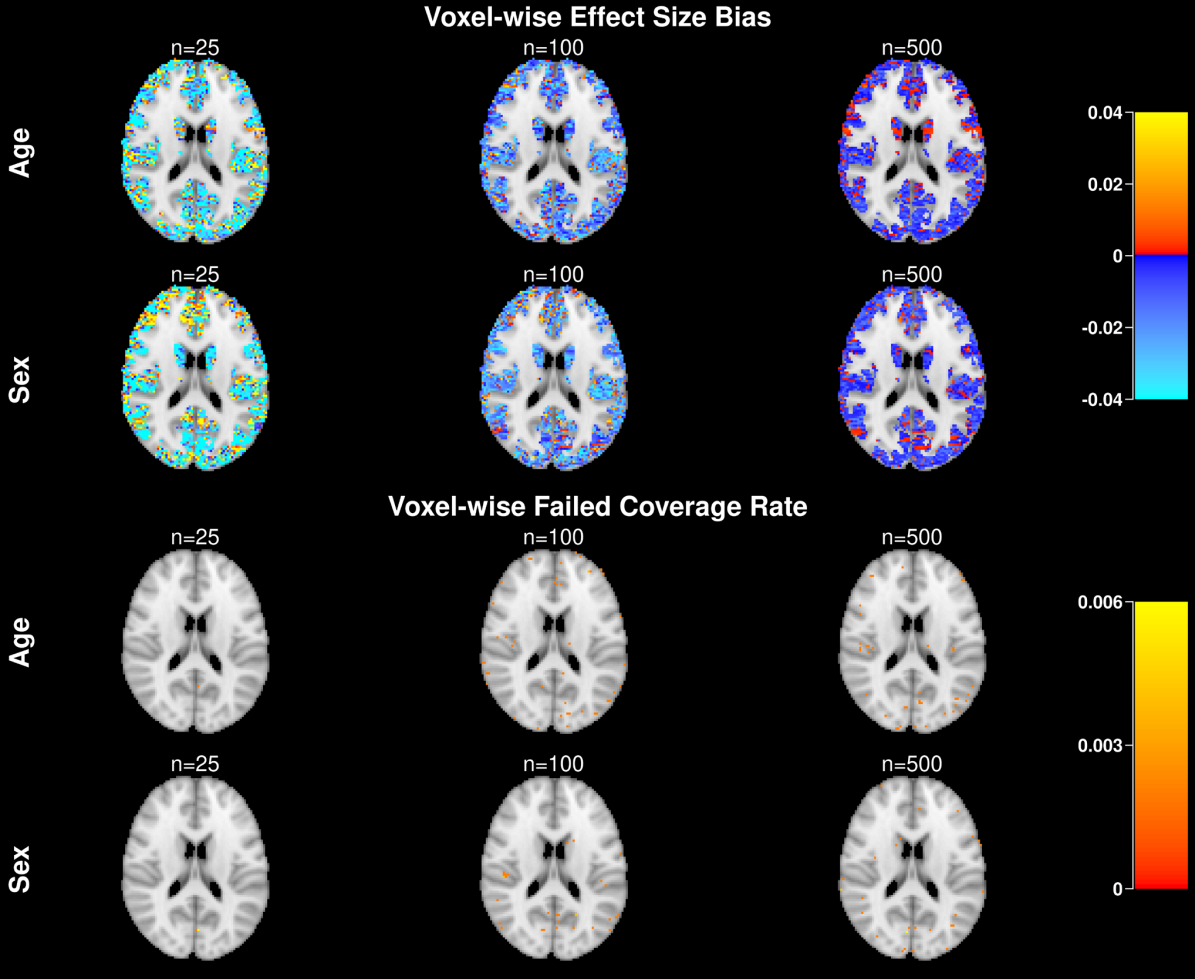
Voxel-wise bias of the effect size estimator (top) and voxel-wise failed coverage rate (bottom) when the true covariance structure is AR1, the working structure is exchangeable, and the Bootstrap method is percentile-t. The color bar range in the bias plot is determined by computing the voxelwise bias range within each of the six scenarios (three sample sizes × two covariates), and then taking the median of the lower bounds and the median of the upper bounds across all scenarios. The color bar range below is set from 0 to the maximum voxelwise failed coverage rate observed across all six scenarios.

We also evaluate spatial patterns of bias of the effect size estimator at the voxel-level (Fig. 2, top). As expected, the bias decreases as the sample size increases. Finally, we evaluate voxel-wise coverage of the percentile-t bootstrap to ensure that there are no spatial patterns of false positives (Fig. 2, bottom). False positives are rare and appear randomly throughout the image plane. Because the procedure controls simultaneous coverage at the 0.95 level across all voxels, the voxel-wise coverage is necessarily much higher than 0.95, which explains why the observed voxel-wise failure rates are well below 0.05 and range from 0 to 0.006 in our simulations.

To evaluate the computational burden of the proposed method under different correlation structures, we record the average computation time to complete one simulation analysis under each scenario (Table 3). See Supplementary Figure S3 for the relative efficiency of the age effect.

**Table 3.** Average computation time (in seconds) for one simulation under different correlation structures and sample sizes.

n	True	Working: independent	Working: exchangeable
25	Independent	45.152	44.657
	Exchangeable	45.266	44.314
	ar1	45.663	45.834
100	Independent	53.699	50.575
	Exchangeable	52.133	52.685
	ar1	51.602	52.635
500	Independent	195.727	274.961
	Exchangeable	207.922	269.736
	ar1	213.023	264.686

## 5 Data Analysis

### 5.1 Cortical thickness in Alzheimer’s disease

**Fig. 3.**
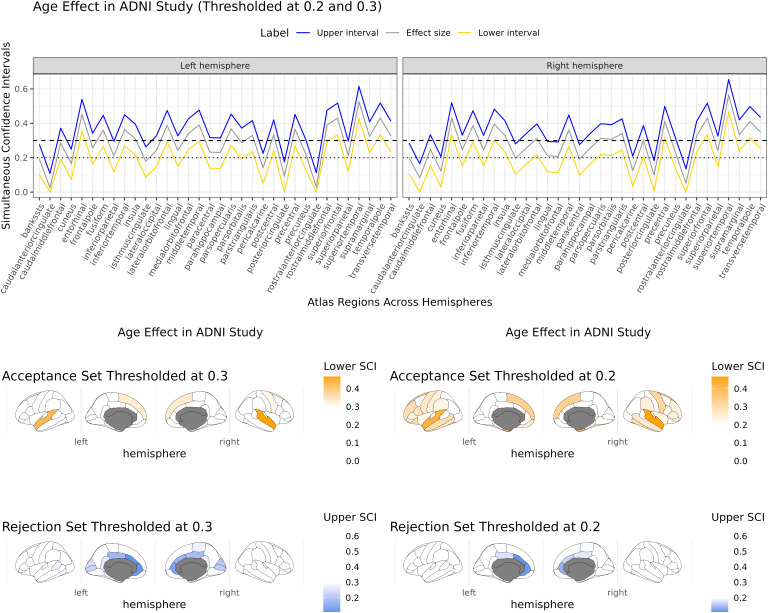
Simultaneous confidence intervals (top) and confidence sets (bottom) for the associations of cortical thickness with age RESIs across all atlas regions in the left and right hemispheres. The lower confidence limit (yellow) is used to construct an inner confidence set using the threshold s=0.3 (dashed line) and s=0.2 (dotted line), which identifies regions of the brain where the true effect size is likely to be larger than that value. The upper confidence limit (blue) is used to construct the rejection set using the same threshold, identifying regions where the effect size is likely to be smaller than that value (Section 3.4).

We use the proposed methods to construct confidence sets of the RESI for associations of age and the five diagnostic groups in the longitudinal ADNI dataset within the Desikan-Killany atlas (Desikan et al., 2006). In this analysis, the spatial index v corresponds to one of the 68 atlas regions (34 per hemisphere), rather than individual voxels. We fit cortical thickness in each region, including sex, a natural cubic spline term for age with 3 degrees of freedom, a 62-level factor variable for the site, and a five-level factor variable for the group with an exchangeable working correlation structure. We visualize regions in the “rejection set”, ${\left\{{\text{CS}}_{\text{out}}\left(s,\in \right)\right\}}^{C}$, where the effect size is likely to be smaller than a given threshold, s, in blue/cyan and regions in the acceptance set, ${\text{CS}}_{\text{in}}\left(s,\in \right)$, in red/yellow. The lower simultaneous confidence limit is used to construct the acceptance set, and the upper confidence limit is used to construct the rejection set (Fig. 3).

We calculated the RESI for the age and diagnosis effects corresponding to each atlas region along with the SCIs. We chose s=0.3 and s=0.2 as the threshold corresponding to approximately “medium” effect sizes using Cohen’s recommendations (Cohen, 1988). We use the confidence sets to identify regions whose association is stronger than these thresholds with 95% confidence (in the Frequentist sense) by thresholding the lower limit, and regions with effect sizes smaller than this threshold using the upper confidence limit. Because the confidence sets are simultaneous, we can apply both thresholds and still maintain the same error rate (Telschow et al., 2024).

For age, the superior temporal lobe, superior frontal lobe, and entorhinal cortex have effect sizes larger than 0.3, indicating at least medium strength associations of cortical thickness with natural aging within these regions (Fig. 3, bottom, “acceptance set”). Regions in the anterior cingulate cortex, occipital lobe, and motor cortex are identified as being in the rejection set (using s=0.3 threshold), identifying where the effect size is less than a “medium” effect size. Both statements are made with a simultaneous 95% family-wise error rate over all regions and any thresholds. Notably, most of the SCIs do not contain zero, indicating that we would reject the null hypothesis in most regions. The confidence set approach allows us to identify important regions based on their effect size instead of the hypothesis test.

**Fig. 4.**
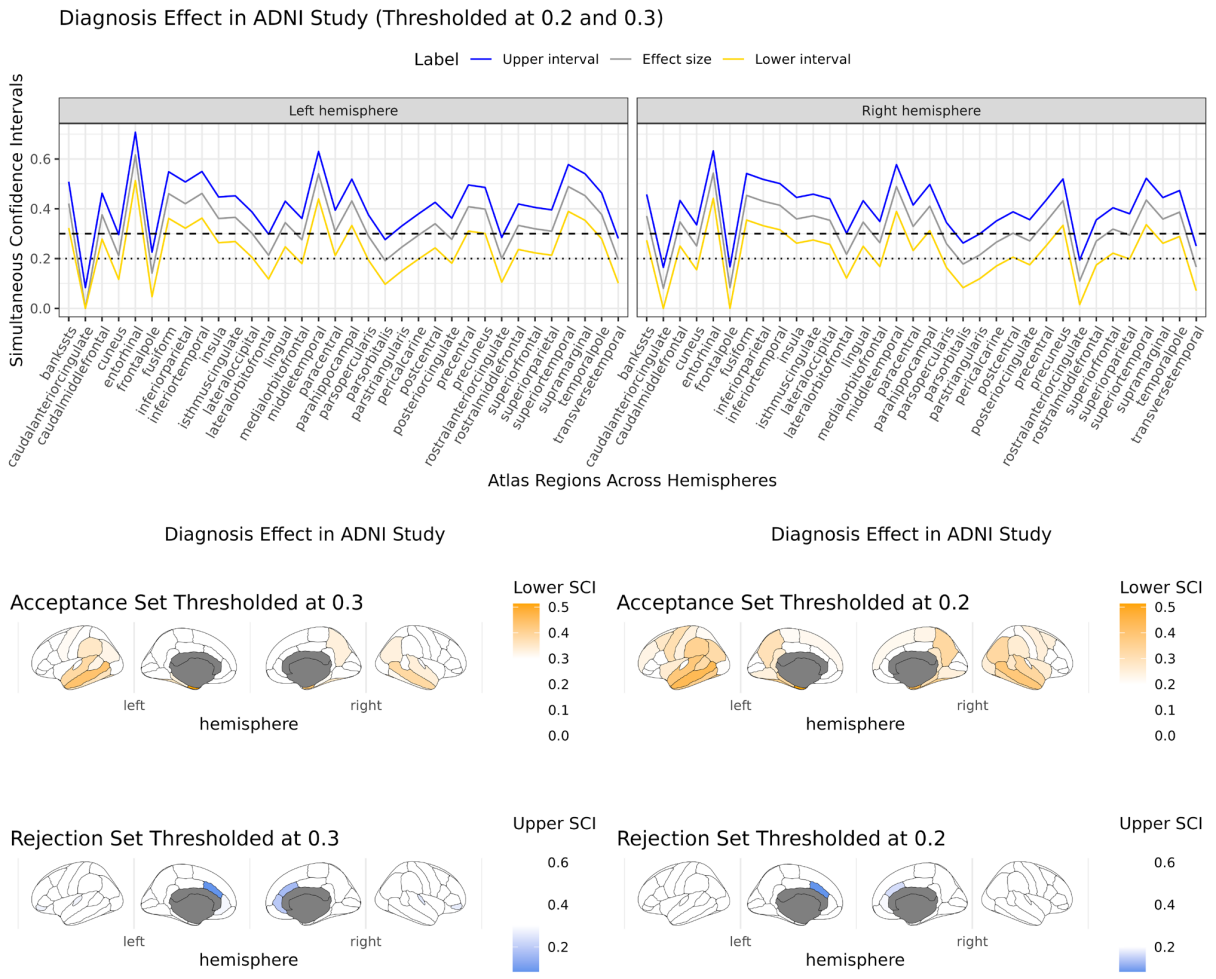
Simultaneous confidence intervals (top) and confidence sets (bottom) for the associations of cortical thickness with diagnosis RESIs across all atlas regions in the left and right hemispheres. The lower confidence limit (yellow) is used to construct an acceptance set using the threshold s=0.3 (dashed line) and s=0.2 (dotted line), which identifies regions of the brain where the true effect size is likely to be larger than that value. The upper confidence limit is used to construct the rejection set using the same threshold, identifying regions where the effect size is likely to be smaller than that value (Section 3.4).

For diagnosis, we identify temporal, parietal, and motor regions with effect sizes larger than s=0.3 , with the strongest effect sizes in middle temporal, superior temporal, and entorhinal cortex (Fig. 4, bottom, “acceptance set”). These regions with the strongest effect sizes align with the wide body of research implicating the temporal lobe in memory decline and AD (Chan et al., 2001; Visser et al., 1999). We defined a rejection set using the upper interval of 0.3, capturing regions in the ACC and inferior frontal lobe. Although the rejection set includes only a small number of regions, it identifies areas where the upper bound of the effect size is below the “medium” threshold (0.3), indicating that these regions are very likely to have effects smaller than a “medium” effect size. Taken together, these results suggest the largest diagnostic effects are isolated in the temporal and parietal lobes. As with the age effect, most simultaneous intervals do not include zero, indicating that adjusted hypothesis tests would identify most regions as significant. The confidence set approach allows investigators to identify regions as important based on their effect size instead. The computation time for this study was 46.56 min for the age effect and 47.11 min for the diagnosis effect, based on 1,709 subjects and 5,604 total observations using 1000 bootstraps across 68 left and right hemisphere atlas regions (34 for each).

### 5.2 Differences in fALFF in early psychosis

We investigated the association between early psychosis and fALFF in the hippocampus using the proposed methods to construct confidence sets for the effect size of group differences (healthy control vs. early psychosis) at all voxels in the hippocampus. The model is adjusted by time (t0 = study entry, t2yr = 2-year follow-up), sex, race, and age fit with natural cubic splines on 3 degrees of freedom. Repeated measurements within a participant are modeled with an exchangeable working correlation structure.

**Fig. 5.**
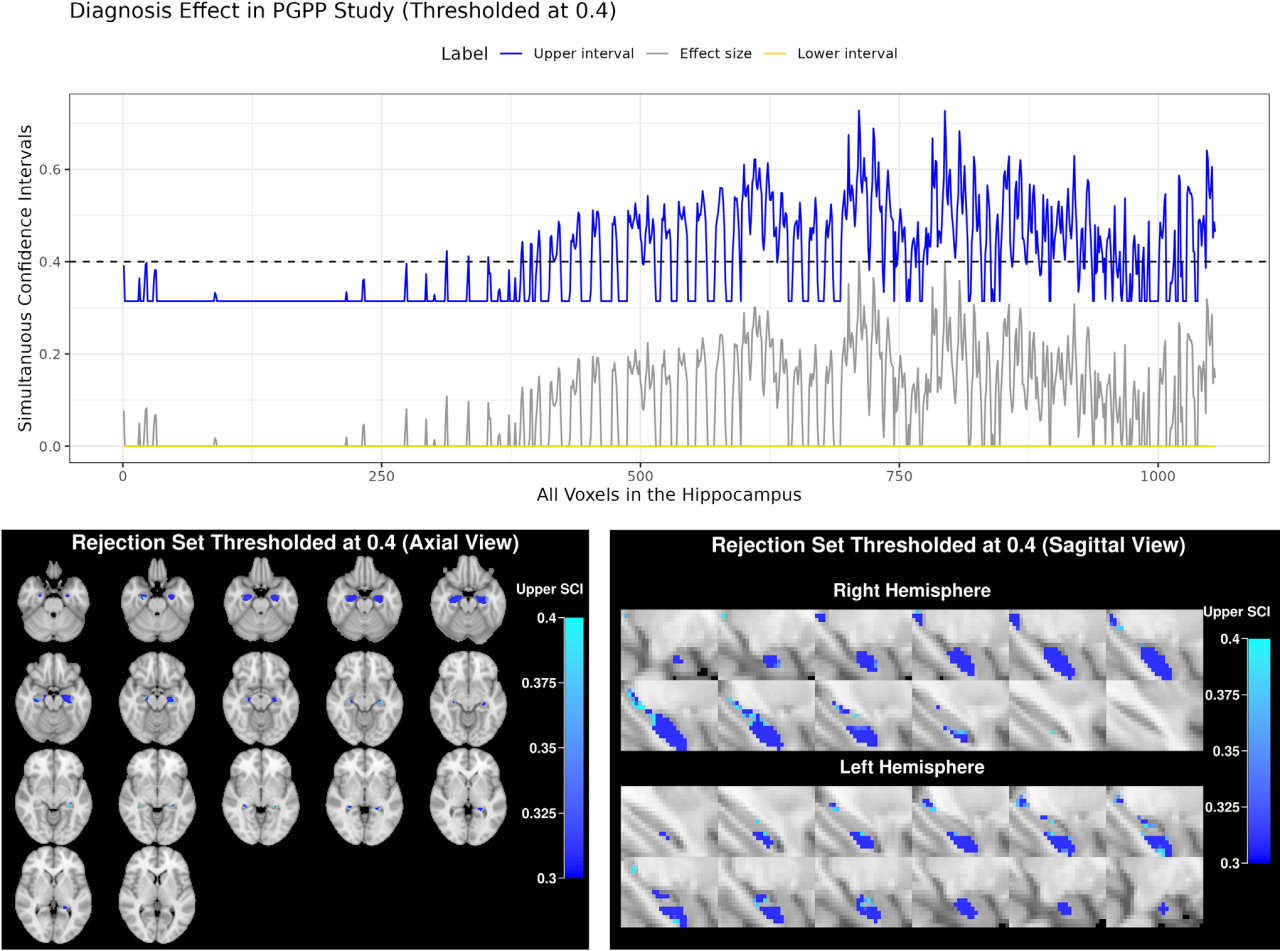
Simultaneous confidence intervals and rejection sets for the voxelwise associations between fALFF and diagnosis RESIs across the hippocampus. The top panel shows simultaneous confidence intervals across all voxels, with the upper limit (blue) compared to a threshold of s=0.4 (dashed line).The rejection sets are visualized in axial (bottom left) and sagittal (bottom right) views, identifying regions where the true effect size is unlikely to exceed 0.4. For the axial view, slices 23-39 are shown; For the sagittal view, slices 28–39 (right hemisphere) and 53–64 (left hemisphere) are shown. The color variation reflects different levels of effect size: darker blue indicates smaller effect sizes, while light blue corresponds to larger effect sizes that still fall below 0.4 with 95% confidence.

The SCIs for the association of early psychosis with fALFF show no voxels have a lower confidence limit above zero (Fig. 5, top), suggesting that there are limited regions where we can confidently conclude an effect of early psychosis on hippocampal E/I imbalance. Thresholding the upper simultaneous confidence limit below 0.4 identifies regions where the effect size is likely to be smaller than a “large” effect size (Cohen, 1988; S. N. Vandekar et al., 2019) (Fig. 5, bottom). One strength of this approach is the potential to identify regions with effect sizes smaller than 0.4 (shown in blue); this can be used to provide sample size justification for future study design by estimating a strict minimum sample size needed for a given target power. Alternatively, measures of fALFF could be improved using longer acquisition times or repeated acquisitions to increase effect sizes for the association. As above, the use of simultaneous intervals allows us to visualize the confidence sets at multiple thresholds without increasing the error rate. The computation time for this analysis was 33.5 seconds, based on 126 subjects and 228 total observations using 1000 bootstraps.

The bounded shape of the upper SCI in Figure 5 arises because the effect size estimator ${\hat{S}}_{\beta }\left(v\right)$ is bounded below by 0 (see Equation (7)). When the observed estimates are near this lower bound, the bootstrap distribution becomes truncated, which limits the variability and leads to a flat SCIs.

## 6 Discussion

We used recently developed statistical methods to develop an approach to construct confidence sets for the RESI in cross-sectional and longitudinal analyses and used the methods to study diagnostic associations in Alzheimer’s disease with cortical thickness and associations of psychosis-spectrum diagnosis with resting-state activity in the hippocampus. Our methods address a gap in confidence set inference for multiparameter statistics, such as nonlinear age effects and multi-group diagnoses in longitudinal models in neuroimaging (Bowring et al., 2019, 2021). Using simultaneous confidence intervals allows the user to construct confidence sets across all possible effect size thresholds while still maintaining 95% coverage (Maullin-Sapey et al., 2024; Telschow et al., 2024).

A critical limitation of the method is that the width of the intervals can be wide for small studies. Using the simultaneous intervals is similar to controlling the voxel-wise family-wise error rate using hypothesis testing, which can lead to conservative results. For example, in the PGPP study, which included 126 participants, the intervals were too large to identify regions in the acceptance set. This limitation is noted by Bowring et al. (2021) when constructing confidence sets for Cohen’s d. For this reason, the confidence set approach may be better suited for larger neuroimaging studies where effect size estimation is the primary objective. Potentially, meta-analytic methods can be used to aggregate effect estimates across studies to build tighter confidence sets. It may also be possible to combine our work with theoretically optimal methods for constructing simultaneous confidence intervals (Gao et al., 2021) to construct tighter confidence sets. In addition, developing methods of confidence sets for a single, fixed threshold, or that control the equivalent of the false discovery rate may lead to tighter confidence sets (Ren et al., 2024; Telschow et al., 2024).

In conclusion, the tool we developed leverages recent statistical methods for confidence sets to perform inference in neuroimaging with multi-dimensional parameters and longitudinal data, expanding the research questions that can be studied using confidence sets. With the code available in the pbj R package, these methods can improve effect size reporting in neuroimaging. The statistical framework is general and can accommodate different spatial units of analysis, including voxel-wise data and atlas-based parcellations. Here, we accommodated atlas-based analysis by converting the input data to niftiImage object (see Data and Code Availability section).

## Supplementary Material

Supplementary Material

## Data Availability

The ADNI Data used in this article were obtained from the Alzheimer’s Disease Neuroimaging Initiative (ADNI) database (adni.loni.usc.edu/). The PGPP data are not publicly available. The methods presented in this paper are provided in the pbj R package (https://github.com/statimagcoll/pbj), and the code for analyses can be found here (https://github.com/statimagcoll/Confidence_Sets.git).
