# Supplementary material for: Semiparametric confidence sets for cross-sectional and longitudinal neuroimaging
Source: Imaging Neurosci (Camb). 2025 Oct 31;3:IMAG.a.965. doi: 10.1162/IMAG.a.965 (PMC12580815; doi:10.1162/IMAG.a.965)
Supplement: Supplementary Material [file IMAG.a.965_supp.pdf]

Supplement for "Semiparametric Confidence Sets for Cross-sectional and Longitudinal Neuroimaging"

Xinyu Zhang,[1,†,*] Kenneth Liao,[1,†] Jakob Seidlitz,[2] Maureen McHugo,[3]
Suzanne N. Avery,[4] Anna Huang,[4] Aaron Alexander-Bloch,[2]
Neil Woodward,[4] Stephan Heckers,[4] and Simon Vandekar[1,5,*]

[1]Department of Biostatistics, Vanderbilt University,

[2]Department of Psychiatry, University of Pennsylvania,

[3]Department of Psychiatry, University of Colorado Anschutz Medical Campus,

[4]Department of Psychiatry and Behavioral Sciences, Vanderbilt University Medical Center,

[5]Department of Biostatistics, Vanderbilt University Medical Center

[†]These authors contributed equally to this work

[*]Correspondence: xinyu.zhang@vanderbilt.edu, simon.vandekar@vumc.org

October 6, 2025

S1 Generalized Estimating Equation

S1.1 Derivation of the estimating equation

The estimator and covariance for $\beta(v)$ are derived as the score equation under an exponential family model using the identity link function, $h(X_i\zeta(v)) = X_i\zeta(v)$ (Liang & Zeger, 1986). This approach is equivalent to maximum likelihood estimation for model (1) with known variance, assuming $E_i(v)$ is multivariate normal (Liang & Zeger, 1986). Specifically, we begin with the general GEE in Liang and Zeger, 1986, Equation (6):

$$\sum_{i=1}^{n} D_i^\top V_i^{-1} S_i = 0,$$

where $S_i = Y^{(i)} - \mu^{(i)}$, $D_i = \frac{\partial \mu^{(i)}}{\partial \zeta}$, and V_i is the working covariance for subject i. In our voxel-wise model, for each voxel v, we consider: $Y_i(v) = X_i\zeta(v)+E_i(v)$, where X_i is the design matrix for subject i, $\zeta(v)$ is the voxel-specific parameter vector, and $\mathrm{Cov}\{E_i(v), E_i(w)\} = \Sigma_i(v, w)$. Then, $\mu_i(v) = X_i\zeta(v)$ and $D_i = X_i$. Substituting into the GEE, we obtain:

$$\sum_{i=1}^{K} X_i^T V_i^{-1} \left[Y_i(v) - X_i\zeta(v)\right] = 0.$$

Combining all individuals into one matrix gives the equation for Ψ in Equation (2) in the paper:

$$\Psi(\zeta(v); Y) = X^\top V_w^{-1} \left[Y(v) - X\zeta(v)\right] = 0.$$

S1.2 Estimator for $\beta(v)$

Solving the above equation for $\zeta(v)$ yields,

$$\hat{\zeta}(v) = (X^T V_w^{-1} X)^{-1} X^T V_w^{-1} Y(v). \tag{S1}$$

The estimator for $\hat{\beta}(v)$ is obtained by simplifying the terms for $\hat{\beta}(v)$ in (S1). Specifically,

$$\hat{\zeta}(v) = (X^T V_w^{-1} X)^{-1} X^T V_w^{-1} Y(v) = \begin{bmatrix} X_0^T V_w^{-1} X_0 & X_0^T V_w^{-1} X_1 \\ X_1^T V_w^{-1} X_0 & X_1^T V_w^{-1} X_1 \end{bmatrix}^{-1} \begin{bmatrix} X_0^T V_w^{-1} Y(v) \\ X_1^T V_w^{-1} Y(v) \end{bmatrix},$$

then by Bernstein, 2009, proposition 2.8.7 we obtain the block components of the inverse matrix

$$\hat{\beta}(v) = \left(X_1^T V_w^{-1} X_1 - X_1^T V_w^{-1} X_0^\top (X_0^T V_w^{-1} X_0)^{-1} X_0^T V_w^{-1} X_1\right)^{-1} \left(X_1^\top - X_1^T V_w^{-1} X_0^\top (X_0^T V_w^{-1} X_0)^{-1} X_0^\top\right) V_w^{-1} Y.$$

Let $\tilde{X}_0 = V_w^{-1/2} X_0$, $\tilde{X}_1 = V_w^{-1/2} X_1$ and $\tilde{Y} = V_w^{-1/2} Y$, substituting into the above expression:

$$\hat{\beta}(v) = \left(\tilde{X}_1^\top \tilde{X}_1 - \tilde{X}_1^\top \tilde{X}_0 (\tilde{X}_0^\top \tilde{X}_0)^{-1} \tilde{X}_0^\top \tilde{X}_1\right)^{-1} \left(\tilde{X}_1^\top - \tilde{X}_1^\top \tilde{X}_0 (\tilde{X}_0^\top \tilde{X}_0)^{-1} \tilde{X}_0^\top\right) \tilde{Y}.$$

By factoring out the common structure, we identify:

$$R_0 = I - \tilde{X}_0 (\tilde{X}_0^\top \tilde{X}_0)^{-1} \tilde{X}_0^\top \quad \Rightarrow \quad \hat{\beta}(v) = \left(\tilde{X}_1^\top R_0 \tilde{X}_1\right)^{-1} \tilde{X}_1^\top R_0 \tilde{Y}$$

Finally, we can express the estimator back in terms of the original variables:

$$R_0 = I - V_w^{-1/2} X_0 (X_0^\top V_w^{-1} X_0)^{-1} X_0^\top V_w^{-1/2}$$
$$\hat{\beta}(v) = \left(X_1^\top V_w^{-1/2} R_0 V_w^{-1/2} X_1 \right)^{-1} X_1^\top V_w^{-1/2} R_0 V_w^{-1/2} Y$$

Similarly, we have

$$R = I - V_w^{-1/2} X (X^\top V_w^{-1} X)^{-1} X^\top V_w^{-1/2}.$$

The covariance $\Sigma_\beta(v, w)$ is estimated by

$$\hat{\Sigma}_\beta(v, w) =$$
$$(X^T V_w^{-1/2} X)^{-1} \sum_{i=1}^{n} \left[X_i^T V_w^{-1/2} R_{i,i}^{-1} \left\{ R V_w^{-1/2} Y(v) \right\}_i \left\{ R V_w^{-1/2} Y(w) \right\}_i^T R_{i,i}^{-1} V_w^{-1/2} X_i \right] (X^T V_w^{-1/2} X)^{-1}. \tag{S2}$$

Bell and McCaffrey, 2002 first proposed this jackknife estimator for multi-stage data, which was later referred to as the CV_3 estimator by MacKinnon et al., 2023. This class of estimators, based on cluster-level residual projections and leverage adjustment, yields more accurate inference than conventional sandwich estimators, particularly in the presence of few clusters or high leverage variability (MacKinnon et al., 2023). Here we ignore the factor $\frac{n-1}{n}$ as it is asymptotically negligible.

S2 Working Covariance Structure

Let $V_{w,i}(\gamma) = \left(A_i\right)^{1/2} C_{w,i}(\gamma) \left(A_i\right)^{1/2} \in \mathbb{R}^{n_i \times n_i}$ represent the working covariance matrix for the i-th subject. Here, $\left(A_i\right)^{1/2} = \mathrm{diag}\{\sigma_i\}$ contains the standard deviations of Y_i, and $C_{w,i}(\gamma)$ denotes the working correlation matrix of the n_i repeated measures, parameterized by γ. Computing $\left(V_{w,i}(\gamma)\right)^{-1}$ requires inverting $C_{w,i}(\gamma)$, which can be computationally challenging.

S2.1 The Exchangeable Structure

The exchangeable correlation structure for the i-th subject is defined as

$$C_{w,i}(\gamma) = \begin{bmatrix} 1 & \gamma & \cdots & \gamma \\ \gamma & 1 & \cdots & \gamma \\ \vdots & & \ddots & \vdots \\ \gamma & \cdots & \gamma & 1 \end{bmatrix},$$

and we assume all subjects share the same correlation parameter γ. Following Example 3 in Liang and Zeger, 1986, the parameter γ can be estimated using the Pearson residuals by

$$\begin{aligned} \hat{\gamma} &= \left\{ \sum_{i=1}^{n} \frac{n_i(n_i-1)}{2} - m_1 \right\}^{-1} \sum_{i=1}^{n} \sum_{j<k} \hat{e}_{ij}\hat{e}_{ik} \\ &= \left\{ \sum_{i=1}^{n} \frac{n_i(n_i-1)}{2} - m_1 \right\}^{-1} \sum_{i=1}^{n} \frac{\left(\sum_{j=1}^{n_i} \hat{e}_{ij} \right)^2 - \sum_{j=1}^{n_i} \left(\hat{e}_{ij} \right)^2}{2}, \end{aligned}$$

where m_1 is the number of covariates in X_1, n is the number of subjects, and n_i is the number of repeated measures for subject i.

The Pearson residuals $\hat{e}_{ij}$ for the j-th measurement of the i-th subject are defined as

$$\hat{e}_{ij} = \frac{Y_{ij} - \hat{Y}_{ij}}{\hat{\sigma}_i},$$

where $\hat{Y}_{ij}$ is the fitted value for Y_{ij}, and $\hat{\sigma}_i$ is the estimated standard deviation for subject i, computed as

$$\hat{\sigma}_i = \left\{ \frac{\sum_{j=1}^{n_i} \left(Y_{ij} - \hat{Y}_{ij} \right)^2}{n_i - m_1} \right\}^{1/2}.$$

Then the inverse of $C_{w,i}(\hat{\gamma})$ can be written as (Qu et al., 2000)

$$\left\{ C_{w,i}(\hat{\gamma}) \right\}^{-1} = \frac{1}{1-\hat{\gamma}} \cdot I_{n_i} - \frac{\hat{\gamma}}{(1-\hat{\gamma})(1+(n_i-1)\hat{\gamma})} \cdot J_{n_i} = aI_{n_i} + bJ_{n_i},$$

where I_{n_i} is a $n_i \times n_i$ identity matrix, J_{n_i} is a matrix of one with size n_i,

$$a = \frac{1}{1-\hat{\gamma}},$$

$$b = -\frac{a\hat{\gamma}}{1 + (n_i - 1)\hat{\gamma}}.$$

To simplify notation, we omit the explicit dependence of C_w on $\hat{\gamma}$, a, and b in the equations below. To efficiently compute $\big(C_{w,i}\big)^{-1/2}$, we derive its explicit expression. First, consider the eigendecomposition of $\big(C_{w,i}\big)^{-1}$, given by

$$\big(C_{w,i}\big)^{-1} = U_i D_i U_i^T,$$

where D_i is the diagonal matrix of eigenvalues, and U_i contains the corresponding eigenvectors. The eigenvalues in D_i are a with multiplicity $n_i - 1$, and $a + n_i b$ with multiplicity 1, then $D_i = \mathrm{diag}\left\{\underbrace{a, a, \ldots, a}_{n_i - 1}, a + n_i b\right\}$. The eigenvectors in U_i are derived via the Gram-Schmidt orthogonalization process to ensure they form an orthonormal basis,

$$v_1 = \frac{1}{\sqrt{2}}\begin{bmatrix} 1 \\ -1 \\ 0 \\ 0 \\ \vdots \\ 0 \\ 0 \end{bmatrix}, v_2 = \frac{1}{\sqrt{6}}\begin{bmatrix} 1 \\ 1 \\ -2 \\ 0 \\ \vdots \\ 0 \\ 0 \end{bmatrix}, \cdots, v_{n_i - 1} = \frac{1}{\sqrt{n_i(n_i - 1)}}\begin{bmatrix} 1 \\ 1 \\ 1 \\ 1 \\ \vdots \\ -(n_i - 1) \\ 0 \end{bmatrix}, v_{n_i} = \frac{1}{\sqrt{n_i}}\begin{bmatrix} 1 \\ 1 \\ 1 \\ 1 \\ \vdots \\ 1 \\ 1 \end{bmatrix}.$$

The inverse square root of C_i, denoted as $\big(C_i\big)^{-1/2}$, can then be efficiently computed as:

$$\big(C_i\big)^{-1/2} = U_i \big(D_i\big)^{1/2} U_i^T.$$

Here, $\big(D_i\big)^{1/2}$ is the diagonal matrix whose entries are the square roots of the reciprocals of the eigenvalues in D_i.

S3 Asymptotic Variance of RESI Estimator

Under the normality of data, for the model $Y = X\beta + \epsilon$, where $\beta \in \mathbb{R}^{m_1}$, $Y \in \mathbb{R}^n$ and $\epsilon \sim N(0, \sigma^2 V)$ we have:

$$T_{m_1}^2 = (\hat{\beta} - \beta_0)^T \hat{\Sigma}_\beta^{-1} (\hat{\beta} - \beta_0)$$

$$\frac{T_{m_1}^2}{m_1} = \frac{\chi_{m_1}^2(nS_\beta^2)/m_1}{\chi_{n-m_1}^2/(n-m_1)} \sim F_{m_1,n-m_1}(nS_\beta^2)$$

In the paper, the estimator of the effect size used to derive the asymptotic variance is given by

$$n\tilde{S}_\beta^2(v) = T_{m_1}^2(v).$$

The degrees of freedom (DF) of the model (1) is $n - m$, so the second DF in the above noncentral F statistics will be replaced by $n - m$ for the following computation. Plugging it into the effect size estimator, we obtain

$$\sqrt{n}\tilde{S}_\beta^2(v) \sim \frac{m_1}{\sqrt{n}} \cdot F_{m_1,n-m}(nS_\beta^2(v)).$$

By the variance of the noncentral F distribution, the asymptotic variance of $\sqrt{n}\tilde{S}_\beta^2(v)$ can be derived as

$$\begin{aligned}
\text{Var}\left\{\sqrt{n}\tilde{S}_\beta^2(v)\right\} &= \text{Var}\left\{\frac{m_1}{\sqrt{n}}F_{m_1,n-m}(nS_\beta^2(v))\right\} \\
&= \frac{m_1^2}{n} \cdot \text{Var}\left\{F_{m_1,n-m}(nS_\beta^2(v))\right\} \\
&= \frac{m_1^2}{n} \cdot 2 \cdot \frac{\left(m_1 + nS_\beta^2(v)\right)^2 + \left(m_1 + 2nS_\beta^2(v)\right)(n-m-2)}{(n-m-2)^2(n-m-4)} \left(\frac{n-m}{m_1}\right)^2 \\
&= \frac{2(n-m)^2}{n} \cdot \frac{\left(m_1 + nS_\beta^2(v)\right)^2 + \left(m_1 + 2nS_\beta^2(v)\right)(n-m-2)}{(n-m-2)^2(n-m-4)}
\end{aligned}$$

When $n \to \infty$,

$$\lim_{n\to\infty} \text{Var}\left\{\sqrt{n}\tilde{S}_\beta^2(v)\right\} = \lim_{n\to\infty} \frac{2n^2}{n} \cdot \frac{n^2 S_\beta^4(v) + 2n^2 S_\beta^2(v)}{n^2 \cdot n} = 2S_\beta^4(v) + 4S_\beta^2(v)$$

By the univariate delta method (Van der Vaart, 2000, Chapter 3), with transformation $g(x) = \sqrt{x}, \quad g'(x) = \frac{1}{2\sqrt{x}}$, applied to $\tilde{S}_\beta(v) = g(\tilde{S}_\beta^2(v))$, the asymptotic variance of $\sqrt{n}\tilde{S}_\beta(v)$ is

$$\lim_{n\to\infty} \text{Var}\left\{\sqrt{n}\,\tilde{S}_\beta(v)\right\} = \left\{g'\left(S_\beta^2(v)\right)\right\}^2 \cdot \lim_{n\to\infty} \text{Var}\left\{\sqrt{n}\,\tilde{S}_\beta^2(v)\right\}.$$

Substituting $g'\left(S_\beta^2(v)\right) = 1/\left(2S_\beta(v)\right)$, the expression above yields

$$\lim_{n\to\infty} \text{Var}\left\{\sqrt{n}\,\tilde{S}_\beta(v)\right\} = \tfrac{1}{2}\left(S_\beta^2(v) + 2\right).$$

$$\text{Var}\left\{\tilde{S}_\beta(v)\right\} \approx \frac{S_\beta(v)^2/2 + 1}{n}.$$

S4 Asymptotic Covariance Computation

According to the law of total variance,

$$\begin{aligned}
&\lim_{n\to\infty} \mathrm{Cov}\left\{\sqrt{n}\hat{\beta}(v)\right\} \\
&= \lim_{n\to\infty} \mathbb{E}\left\{\mathrm{Cov}\left(\sqrt{n}\hat{\beta}(v) \mid X\right)\right\} + \mathsf{Var}\left\{\mathbb{E}\left(\sqrt{n}\hat{\beta}(v) \mid X\right)\right\} \\
&= \lim_{n\to\infty} \mathbb{E}\left\{\mathrm{Cov}\left(\sqrt{n}\hat{\beta}(v) \mid X\right)\right\} + \mathsf{Var}\left\{\sqrt{n}\beta(v)\right\} \\
&= \lim_{n\to\infty} \mathbb{E}\left\{\mathrm{Cov}\left(\sqrt{n}\hat{\beta}(v) \mid X\right)\right\} \\
&= \lim_{n\to\infty} n\mathbb{E}\left\{(X^T V_w^{-1}(\gamma, v)X)^{-1}\right\}\mathbb{E}\left\{X^T V_w^{-1}(\gamma, v)\Sigma(\rho, v)V_w^{-1}(\gamma, v)X\right\}\mathbb{E}\left\{(X^T V_w^{-1}(\gamma, v)X)^{-1}\right\} \\
&= \lim_{n\to\infty} n\mathbb{E}\left\{(X^T C_{work}^{-1}(\gamma)X)^{-1}\right\}\mathbb{E}\left\{X^T C_{work}^{-1}(\gamma)C_{true}(\rho)C_{work}^{-1}(\gamma)X\right\}\mathbb{E}\left\{(X^T C_{work}^{-1}(\gamma)X)^{-1}\right\}\sigma^2(v)
\end{aligned}$$

where $\Sigma(\rho, v)$ is the true covariance with correlation parameter ρ, $V_w(\gamma, v)$ is the working covariance with correlation parameter γ. The final equality only follows under the separability of the spatial and temporal covariances, where $C_{true}(\rho)$ is the true correlation and $C_{work}(\gamma)$ is the working correlation.

The above equation shows a general form. Rather than assuming a common correlation γ across all subjects, we estimated a subject-specific working correlation γ_i for each cluster. This approach allows the working correlation matrices to flexibly adapt to individual heterogeneity, while inference for the regression coefficients remains valid under the robust sandwich variance estimator even if these working correlations are misspecified.

1. When the working correlation structure is independent, $E\{\hat{\gamma}_i\} = 0$
2. When both the true and working correlation structures are exchangeable, $E\{\hat{\gamma}_i\} = \rho$
3. When the working correlation structure is exchangeable while the true is independent or AR1, see derivation below

We can express the general moment-based estimator for exchangeable working covariance as (Wang, 2003):

$$\sum_{j\neq k} e_{ij}e_{ik} = \sum_{j<k} \gamma_i \left(e_{ij}^2 + e_{ik}^2\right),$$

where e represents standardized residuals $(j, k = 1, \cdots, n_i)$ and n_i is the number of repeated measurements for subject i. The equation then simplifies to:

$$\hat{\gamma}_i = \frac{\sum_{j \neq k} e_{ij} e_{ik}}{(n_i - 1) \sum_{j=1}^{n_i} e_{ij}^2},$$

Assuming that the true temporal correlation structure is independent, the expected value of each pairwise product $\mathbb{E}\{e_{ij} e_{ik}\}$ for $j \neq k$ approaches zero as the sample size increases. Consequently, the numerator in the expression for $\hat{\gamma}_i$ converges to zero.

Assuming the true temporal correlation structure follows an AR1 process, the covariance between two observations Y_{ij} and Y_{ik} at time points j and k $(j, k = 1, \cdots, n_i)$ for subject i is given by $\mathrm{Cov}\{Y_{ij}, Y_{ik}\} = \mathbb{E}\{e_{ij} e_{ik}\} = \rho^{|j-k|}$. The standardized residuals e_{ij} are assumed to have unit variance, i.e., Var $\{Y_{ij}\} = \mathbb{E}\{e_{ij}^2\} = 1$. To estimate the working correlation parameter γ_i under the exchangeable assumption, we use the moment-based estimator:

$$\mathbb{E}\{\hat{\gamma}_i\} = \mathbb{E}\left\{\frac{\sum_{j \neq k} e_{ij} e_{ik}}{(n_i - 1) \sum_{j=1}^{n_i} e_{ij}^2}\right\} = \mathbb{E}\left\{\frac{\sum_{j \neq k} \rho^{|j-k|}}{n_i(n_i - 1)}\right\} = \frac{2}{n_i(n_i - 1)} \sum_{|j-k|=1}^{n_i - 1} (n_i - |j - k|)\rho^{|j-k|}$$

indicating that the estimator $\hat{\gamma}_i$ incorporates the decay of correlation with increasing lag, as expected under the AR1 structure.

S5 Nonseparable Covariance Simulation

We simulate a realistic nonseparable error term as $E_{ij}(v) = Z_i^T R_{s,j}(v)$ for the j-th repeated observation of subject i at location v, where $Z_i \sim N(0, I) \in \mathbb{R}^{N_{PGPP}}$ denote a vector sampled from a standard normal distribution. The spatial residuals $R_{s,j}(v) \in \mathbb{R}^{N_{\text{PGPP}}}$ $(j = 1, 2, 3)$ are deterministically extracted from fixed slices of the PGPP data: $R_{s,1}(v)$ from the 46th slice, $R_{s,2}(v)$ from the 45th slice and $R_{s,3}(v)$ from the 47th slice. Depending on the number of repeated measurements available for subject i, only the corresponding subset of these residuals is used (e.g., if $n_i = 1$, only $R_{s,1}(v)$ is included; if $n_i = 2$, both $R_{s,1}(v)$ and $R_{s,2}(v)$ are included, etc.) while $R_{s,j}(v)$ is always fixed and treated as a column vector. Everything other than the error term is consistent with the simulation setting in the main text, including the model, the coefficients, and the design.

We denote the true within-subject covariance of the repeated error terms as $\Sigma_i(v)$ for subject i at location

v, where each element is given by

$$\Sigma_{i,jk}(v) = \text{Cov}\{E_{ij}(v), E_{ik}(v)\} = \mathbb{E}\left\{R_{s,j}(v)^\top Z_i^\top Z_i R_{s,k}(v)\right\} = R_{s,j}(v)^\top R_{s,k}(v).$$

Since the slice-specific residuals $R_{s,j}(v)$ are fixed and identical across subjects, $\Sigma_i(v)$ does not depend on the subject index beyond the number of repeated measurements n_i. Thus, any two subjects with the same n_i share the same covariance structure.

It breaks the separability assumption by allowing spatial covariance to vary across time points, thereby introducing interactions between space and time that cannot be represented by a Kronecker product used in the paper. The working covariance $V_{w,i}(v)$ is specified as an independent structure, with diagonal elements set to the weighted average of the within-slice variances:

$$\text{diag}\{V_{w,i}(v)\} = \frac{1}{2}R_{s,1}(v)^\top R_{s,1}(v) + \frac{1}{3}R_{s,2}(v)^\top R_{s,2}(v) + \frac{1}{6}R_{s,3}(v)^\top R_{s,3}(v),$$

where the weights are the marginal probabilities of the j-th observation occurring. The true effect size is computed using the same underlying β and the asymptotic covariance $\Sigma_{\sqrt{n}\beta}(v)$, as detailed in the last second line of Section S4. We further compare the performance of the nonseparable and separable models under small sample sizes ($n = 25, 50, 100, 150$), focusing on the simultaneous coverage and the mean width of the simultaneous confidence intervals (multiplied by $\sqrt{n}$). As shown in Figure S1, the simultaneous coverage and SCI widths under the non-separable error structure are comparable to those obtained under separable errors. This finding demonstrates that our method remains robust even when the spatiotemporal correlation deviates from the idealized separable form, highlighting its applicability to more realistic neuroimaging data scenarios.

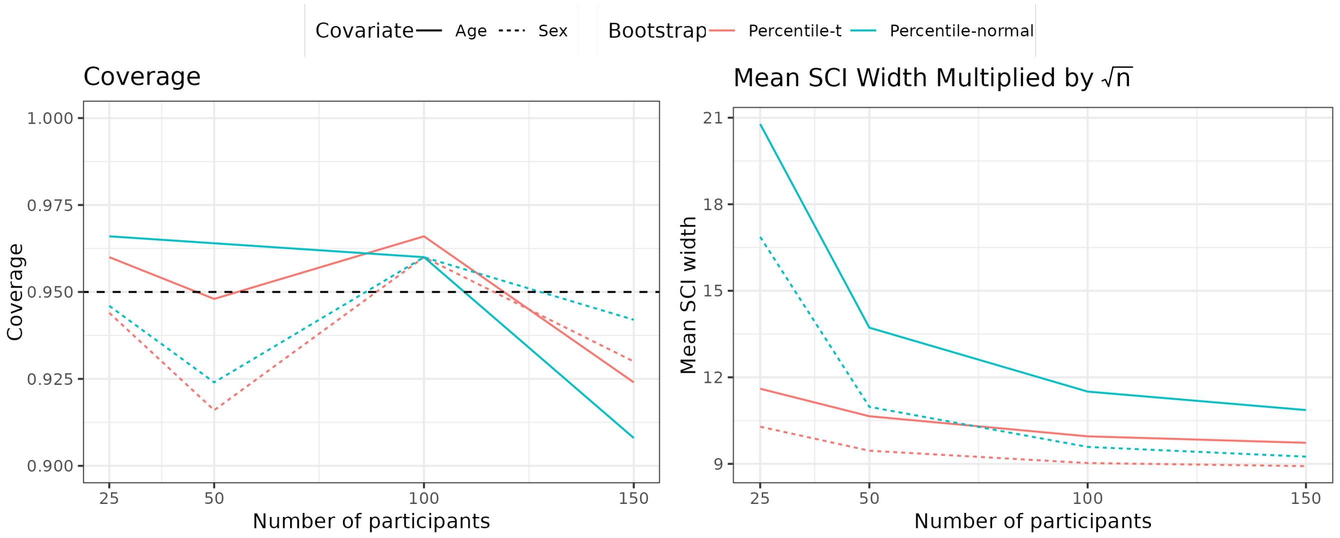

Figure S1: Simulation results for simultaneous coverage (left) and mean width of the simultaneous confidence intervals (right) used to construct confidence sets with non-separable errors. Sample sizes are $n = 25, 50, 100, 150$. There are two covariates (age and sex) and two Bootstrap methods (percentile-t and percentile-normal).

S6 Simulation SCI Plots

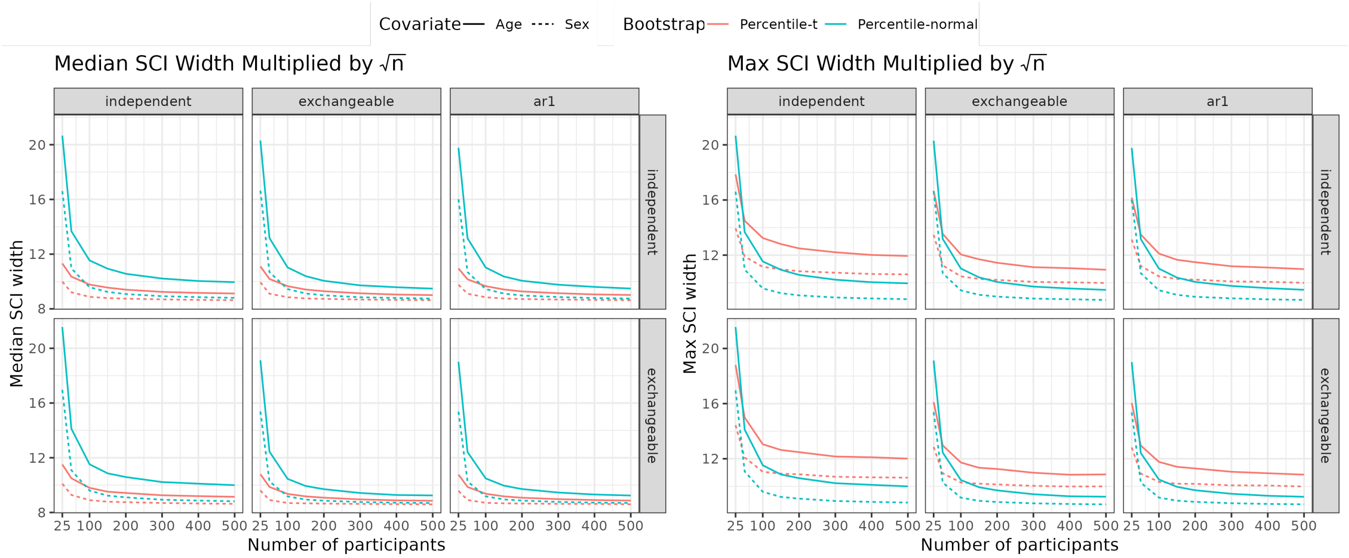

Figure S2: Simulation results for median width (left) and max width (right) of the simultaneous confidence intervals used to construct confidence sets. The panel columns indicate the true covariance structures (independent, exchangeable, and AR1) and working covariance structures (independent and exchangeable) for different sample sizes (25 - 500). Each scenario considers two covariates (age and sex) and two Bootstrap methods (percentile-t and percentile-normal).

S7 Relative Efficiency

Across scenarios, the median of relative efficiency was 1.010 - 1.019 (25th percentile: 0.953 - 0.959; 75th percentile: 1.074 - 1.090), which suggests that the commonly used structures considered (independent and exchangeable) offer reasonable efficiency across a range of true correlation scenarios.

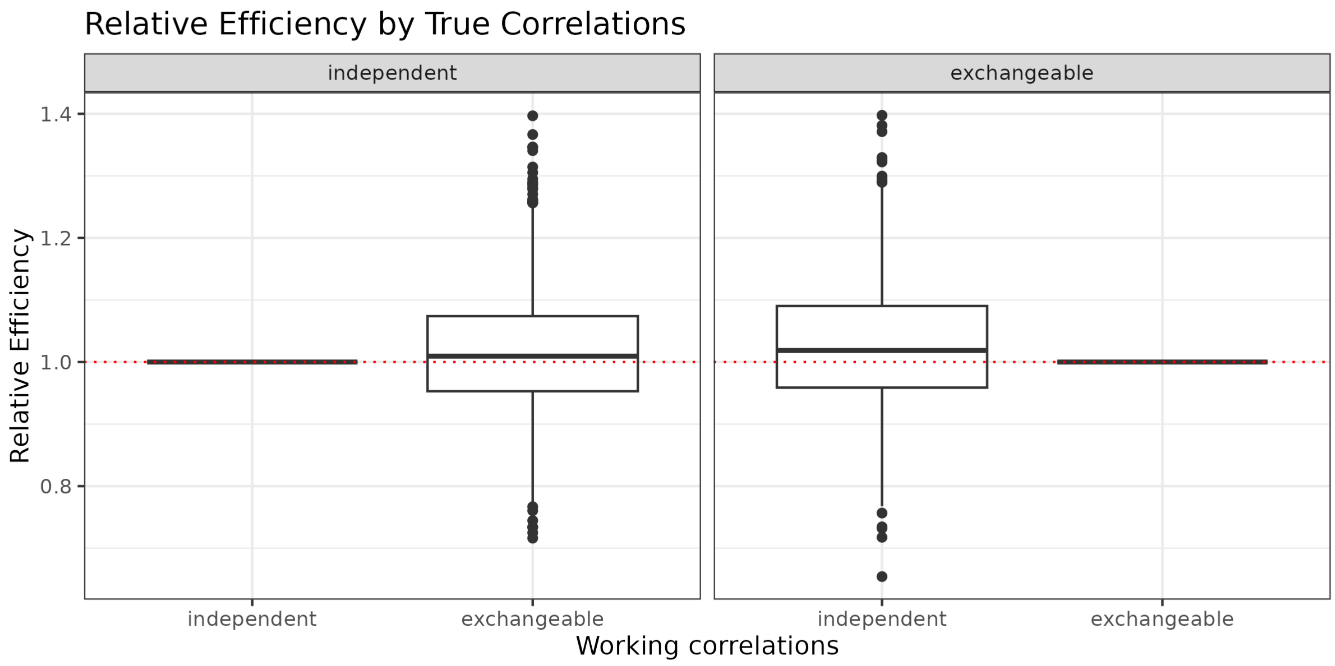

Figure S3: Box plots across voxels of relative efficiency of age effect computed across 500 simulations, under various combinations of true and working correlation structures with a sample size of 400. The Bootstrap method is the percentile-T Bootstrap. For each voxel, we compute its relative efficiency as the ratio of the variance of each estimator across simulations over the variance under correctly specified correlation. Each panel corresponds to a true correlation structure (independent or exchangeable), while the x-axis indicates the working correlation structure (independent or exchangeable). The red dotted line at 1 serves as a reference line representing the correct specification. Note that the relative efficiency is exactly 1 when the working correlation structure matches the true structure, as expected under correct model specification.
